# The Doctor and Infectious Cases

**Published:** 1908-12-12

**Authors:** James Dundas


### The General Practitioner's Column.

[Contributions to this Column are invited, and if accepted will be paid for.]

### THE DOCTOR AND INFECTIOUS CASES.

By JAMES DUNDAS, M.B., D.P.H.

Various duties demanding instant attention fall on the medical man as soon as he finds he has a case of undoubted or supposed infectious disease. Thus, should he find symptoms resembling those of small-pox, which may well be confounded with varicella, or of typhus, he must at once communicate with the medical officer of health of his district, who will almost certainly lose no time in seeing the case with him, and arranging for its isolation, if any doubt remains as to its diagnosis. If epidemics of small-pox and typhus are to be controlled at all, they must be caught early, before the patient has time to disseminate infection broadcast, and as epidemics run up heavy municipal bills, no effort should be spared in securing prompt isolation.

With the more common fevers, such as diphtheria and scarlet, there should be also as little delay as possible in notification. This must be done on the forms provided, but it is better to notify the case at once by telephone, to allow of the early removal of the patient. It is also important, for the sake of the patient, if the case be one of diphtheria, for, as is well known, the earlier serum is exhibited, the better is the prognosis, and as parents are often unwilling to pay for serum, the sooner the patient reaches hospital the better. Remembering too that the need for tracheotomy may be sudden and urgent, admission to hospital secures facilities for carrying this out, and the subsequent nursing of the case without any possibility of delay. Lastly, but by no means least, the hospital staff appreciate the early admission of cases. Rashes and other symptoms are evanescent and often slight, and the sooner they have an opportunity of seeing the case, the more easily and more correctly can it be classified. Nothing is more annoying than to admit a case, say of measles, on the evidence of the history alone, to a scarlet ward, and find out later the true nature of the case, when it breaks out in other patients.

At the same time it is very common for patients suffering from other diseases to have an intercurrent attack of some fever. Frequently these are surgical cases, and a short note indicating what has been done is a courtesy that should not be omitted. Cases of diphtheria, which have had their throats swabbed, or serum administered, should also be accompanied by a note to that effect. Such information is very useful to the hospital officials, and should not be sent by word of mouth *viâ* the removal officer. Besides, such courtesies tend to promote good feeling between both parties.

Before notifying diphtheria and typhoid, many practitioners delay notification until the clinical pathologist is able to confirm the diagnosis. In typhoid this is an excellent practice, as the clinical appearances are so deceptive, and a little delay in isolation is not of much potential danger to contacts, as in the case of other fevers. In diphtheria the same cannot be said. Serum should be administered, and the patient put on a full diphtheria regimen without the bacteriologist's report. If the practitioner has facilities for making film examinations himself, entailing only an hour or two of delay, the practice is advisable, but not otherwise. As often as not the B. Hoffmann, the wrongly-styled pseudo-diphtheria bacillus, will be found, and patients harbouring it should be treated as diphtheritic, thus following the practice of most hospitals.

In the majority of places sanitary authorities have ambulances and removal officers, who are responsible for conveying the patient to hospital, for removing his clothes and bedding to the disinfector and the disinfection of the house. Where this staff is absent, it is the doctor's duty to see that these things are done. He must remember that it is illegal, according to the Public Health Acts, to send such a patient to hospital in a cab or other vehicle, unless he has first notified the owner of the infectious nature of his fare. An infectious person may not be exposed, "wilfully, or without proper precautions," in the streets, in shops, public conveyances, or any public place. It is, therefore, an offence to let a patient walk up to hospital, or to send him there in a cab, or by rail. It is the practitioner's duty to impress this upon parents. Much slackness exists in these matters. Removal officers find children out playing in the street with their companions, and those who should know better allow infectious cases to travel about in a very casual manner.

The doctor's duty is not yet ended, for there are the contacts to be considered. Of these, the other children are the most numerous. They must not return to school until a certain period of quarantine, laid down by the authority, has been accomplished, and they have been duly disinfected, and certified free from disease.

The question of administering serum all round to diphtheria contacts is an important one. The writer has frequently seen a mother and all her children come within a few days of one another to hospital. Had a sufficient dose of serum (1,000 to 2,000 units) been exhibited, this state of things would be obviated. The expense is a stumbling block to this becoming a routine practice, but many sanitary authorities are prepared to supply serum for this purpose, free of charge, and practitioners should not neglect to avail themselves of this advantage.

As regards adults, there are certain occupations which they may not follow until they have been satisfactorily disinfected, and these include milking cows, fruit-picking, or any "other business in such manner as is likely to spread the disease."

It is here that the general practitioner can be most useful to the medical officer of health. He probably knows the circumstances of the family and the employment of each. As their own medical adviser they will listen to what he has to say more sympathetically than to a stranger who has to ferret out information. Frequently it will only be necessary for him to point out the risks of infection and the danger to others, when they will readily fall in with any suggestions he has to make as regards disinfection, isolation, and abstention from work. Should he meet with opposition and the case is urgent he can at once fall back on the medical officer of health, who may be trusted to give him his powerful support; but such an unreasonable attitude he will meet with but rarely.

In working-class families isolation at home is very difficult to carry out. The houses are small, often over-crowded, and the people are not yet educated up to the importance of stringent quarantine. Thus it is far better for cases occurring in these homes to be removed to hospital, and the medical officer of health has powers to enforce this, if in his opinion it is desirable. But in better class houses the patient may well remain at home under the care of his own doctor. He should be isolated in a single room to which no one but the doctor and the nurse may have entry. Outside the door coats or overalls are to be kept for use by the doctor and nurse. The room itself should have its heavy furniture, carpet, and unnecessary curtains, etc., removed before occupancy. All clothing and linen must be steeped in some disinfecting solution in appropriate strength—lysol, izal, or carbolic according to the preference of the physician—prior to removal. Similarly all typhoid excreta should be treated with a like bulk of disinfectant. Linen rags, used in lieu of handkerchiefs, tow, and paper should be burned, and any food taken into the sick-room, and unused, must be destroyed. Dishes should be removed in a bucket of disinfectant solution. With these precautions, and entry to the infected room strictly confined to the doctor and nurse, little fear of spread of the disease need be entertained.

